# Where Have All the Spiders Gone? Observations of a Dramatic Population Density Decline in the Once Very Abundant Garden Spider, *Araneus diadematus* (Araneae: Araneidae), in the Swiss Midland

**DOI:** 10.3390/insects11040248

**Published:** 2020-04-15

**Authors:** Martin Nyffeler, Dries Bonte

**Affiliations:** 1 Department of Environmental Sciences, Section of Conservation Biology, University of Basel, CH–4056 Basel, Switzerland; 2 Department of Biology, Terrestrial Ecology Unit, Ghent University, 9000 Ghent, Belgium; Dries.Bonte@UGent.be

**Keywords:** bottom-up trophic cascade, low abundance, orb-weaving spiders, prey scarcity, western European landscape

## Abstract

Aerial web-spinning spiders (including large orb-weavers), as a group, depend almost entirely on flying insects as a food source. The recent widespread loss of flying insects across large parts of western Europe, in terms of both diversity and biomass, can therefore be anticipated to have a drastic negative impact on the survival and abundance of this type of spider. To test the putative importance of such a hitherto neglected trophic cascade, a survey of population densities of the European garden spider *Araneus diadematus*—a large orb-weaving species—was conducted in the late summer of 2019 at twenty sites in the Swiss midland. The data from this survey were compared with published population densities for this species from the previous century. The study verified the above-mentioned hypothesis that this spider’s present-day overall mean population density has declined alarmingly to densities much lower than can be expected from normal population fluctuations (0.7% of the historical values). Review of other available records suggested that this pattern is widespread and not restricted to this region. In conclusion, the decline of this once so abundant spider in the Swiss midland is evidently revealing a bottom-up trophic cascade in response to the widespread loss of flying insect prey in recent decades.

## 1. Introduction

When the Krefeld Entomological Society, together with colleagues, published their famous long-term study (the “Krefeld study“) in 2017, it became known that the biomass of flying insects had declined by approx. 75% over the past three decades in over 60 nature protection areas of Germany [1]. Another long-term study (the “Munich study“) confirmed the Krefeld results, providing evidence that strong abundance declines of insect populations had occurred in farmland and forests across vast areas of Germany and Switzerland [2]. Similar abundance declines of insect populations have been documented in other long-term studies of other European and North American regions [3,4,5,6,7,8,9]. It is generally accepted that we now live in an era of global insect meltdown [9,10,11,12,13]. This has dramatic ecological implications: the fact that insects comprise the basis of many food chains and food webs [14,15] means that in a world without insects, countless insectivorous species will ultimately become extinct due to starvation [16].

**Figure 1:**
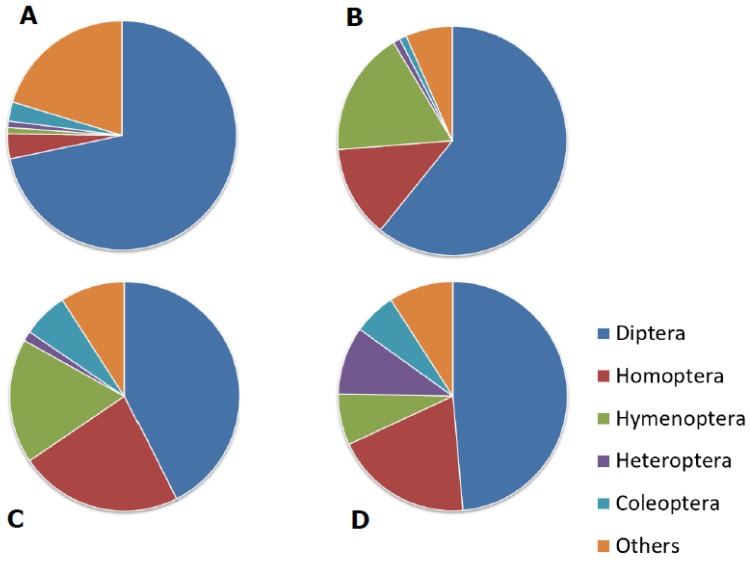
Prey composition (% by number) of the garden orb-weaver *Araneus diadematus* based on four field studies: (**A**) oat field near Zurich, Switzerland [21]; (**B**) fallow grassland near Zurich, Switzerland [21]; (**C**) fallow grassland near Jena, Germany [18]; (**D**) maize field margin near Munich [19]. Homoptera refers primarily to alate aphids.

In this context, aerial web-spinning spiders are a group of utmost interest. These spiders (including the orb-weaving species in the families Araneidae) trap flying insects with the aid of aerial webs [17,18,19]. In the temperate climatic zone, aerial web-spinning spiders feed for the most part on small dipterans, aphids, and hymenopterans (Figure 1), which are exactly the types of insects known to have most dramatically decreased in abundance and biomass in recent decades [1]. Aerial web-spinning spiders, because of their reliance upon flying prey, are therefore a highly vulnerable predator group in regions characterized by a high loss of flying insects, such as Germany and Switzerland.

**Figure 2:**
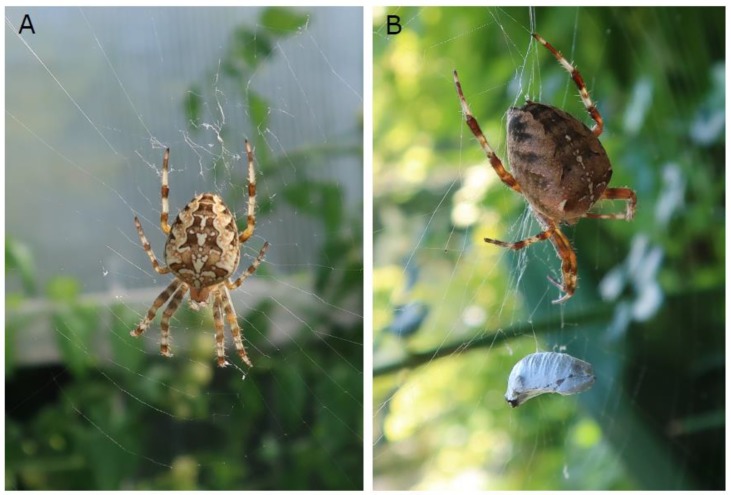
(**A**,**B**) Adult female *Araneus diadematus* in web in a garden in Flawil, northeastern Switzerland (photo by Rätus Fischer).

While a larger number of studies on population declines of various groups of insects has been conducted in recent years (see references in [12]), potential cascading effects have only been documented so far for insectivorous birds [6]. No studies of this type have, to date, considered potential declines due to trophic cascades among arthropods, like aerial web-spinning spiders. Here we used the European garden spider *Araneus diadematus* as a model system to address whether evidence for population declines in aerial web-spinning spiders in the Swiss midland can be found (Figure 2). The female, 10–18 mm in length, reaches adulthood in late summer, at which time it spins (≈30 cm diameter) orbs [20,21,22]. The webs are built 0.5–2 m off the ground [21]. This animal, with its conspicuous white, cross-shaped mark on the upper side of the abdomen, is one of the best known spider species in western Europe.

By comparing historical abundance data (20th century) with present-day data (2019), we examined whether the abundance of *Araneus diadematus* has changed over the past few decades. This work is based on extensive experience gathered during graduate research in the Swiss midland in the 20th century [20,21] and supplemented by a present-day population density survey in the same geographical area. To make the comparison more robust, the data base was expanded by including published population density values extracted from the scientific literature. 

## 2. Materials and Methods 

### 2.1. Assessment of Population Densities of *Araneus diadematus* in 2019

Population density assessments were conducted at 20 locations in the Swiss midland, and the visual censusing technique described by Lubin (1978) [23] was applied at 18 locations. This technique was modified insofar as curved line transects were used in addition to straight line transects in some cases, depending on the paths used [24]. One of us (M.N.) slowly walked along the edge of a path and intensively searched by eye the vegetation within 1 m to one side of the path and from ground level to a height of 2 m (based on the fact that *Araneus diadematus* habitually constructs its webs at heights of 0.5–2 m off the ground [21]). Each adult *Araneus diadematus* web encountered within the transect was recorded. Transects 1 to 5 were 1000 m long, Transects 6 to 10 were 500 m long, and Transects 11 to 18 were 200 m long (see Table S1; transect lengths depended on the nature of the landscape). The search focused on webs of adult females in habitats typical for this species (i.e., gardens, parks, graveyards, hedgerows, forest edges, and forest trails; [25]). Only webs occupied by a spider were counted (regardless whether the spider was present in the web or hiding in a nearby retreat), since only those webs could be counted beyond any doubt as belonging to *Araneus diadematus*. All counts were conducted on rain-free afternoons in August and September 2019. It has been suggested in the literature that spider counts obtained using this method may result in underestimating the true population densities because of the spiders’ cryptic lifestyles [26]. This might well be the case if densities of well-camouflaged, nocturnal, and/or small, immature spiders are included in the assessment [26]. However, in the case of *Araneus diadematus*, a bias of this type is highly unlikely because this is a species of which the adult females construct large, conspicuous webs which are unlikely to be overlooked by a keen observer. Lubin (1978) also stated that “….Visual censusing is an effective technique when applied to animals with conspicuous artifacts, such as most web spiders” [23]. The immature stages of *Araneus diadematus* are more likely to be overlooked with this method because their smaller-sized webs are less conspicuous; however, in the current study this objection was not relevant because immature spiders were not counted.

Additionally, web counts in two organic gardens, covering 330 m^2^ and 800 m^2^ ground area, respectively, were conducted. In these gardens, the vegetation and the exterior of the buildings were thoroughly searched for webs of adult female *Araneus diadematus* on several consecutive days in August and September 2019.

### 2.2. Literature Search for Historical Data on *Araneus diadematus* Population Densities

*Araneus diadematus* population density values assessed in the 1970s in the Zurich area were taken from two graduate research theses [20,21]. These were supplemented by data taken from the literature. Overall, a total of 12 reports (including 18 population density values) were gathered (see Table S1). In these studies, plots ranging from 17.5 to 3300 m^2^ in ground area were used for the assessment of *Araneus diadematus* densities (see Table S1).

It is noticeable that the present habitat distribution was different from the habitats studied in the past, e.g., no fallow grassland was studied in 2019. The fallow grassland areas used in the Swiss studies 40 years ago [21] have fallen victim to urbanization and could not be surveyed. However, *Araneus diadematus* is a habitat generalist, widely distributed over a large number of different habitat types. All the study areas used in the present density assessment (Table 1) represent habitat types in which this species is typically found, such as forested areas [25]. The past century’s spider density values for grasslands were not above that century’s overall mean value (Table 1), and according to Hänggi et al. (1995) [25], tree- and shrub-dominated habitats appear to provide the most suitable sites for construction of the webs of this species. This implies that omission of grassland habitats in the 2019 density assessment should not have compromised our investigation.

### 2.3. Statistical Methods

The data in Table 1 were tested for normality using the Shapiro–Wilk Test Calculator (http://www.statskingdom.com/320ShapiroWilk.html), and this revealed that the data did not follow a normal distribution. The Mann–Whitney *U* test was applied to examine whether spider densities in 2019 (N = 20) differed statistically significantly from those in the 20th century (N = 18). The same test was used to examine whether numbers of prey per web in 2019 and in the 20th century differed statistically significantly. Medians are followed by interquartile ranges (IQR). In the past, multiple estimates of spider densities were probably also not normally distributed but were summarized as a mean. In order to make comparisons with the historical data, we also calculated a mean from our new data (while at the same time acknowledging that this is not the ideal measure).

## 3. Results

In Table 1, population densities of *Araneus diadematus* assessed in the Swiss midland in August/September 2019 are compared with 20th century population density data for this species from a variety of European locations. The table reveals that present-day population densities of adult female *Araneus diadematus* in the Swiss midland were generally extremly low (median = 0.0000 webs m^−2^, IQR = 0.0000–0.0020 webs m^−2^; Table 1). In 2/3 of the 20 investigated transects, no webs of adult female *Araneus diadematus* could be found.

By contrast, historical population densities of *Araneus diadematus* (Figure 2A,B) in its typical habitats were considerably higher (median = 0.0670 webs m^−2^, IQR = 0.0175–0.1835 webs m^−2^; Table 1). The difference between the 20th century European densities and the present-day Swiss densities (median = 0.0670 vs. 0.0000 webs m^−2^) was statistically highly significant (Mann–Whitney *U* test, n_1_ = 18, n_2_ = 20, Z = −4.0637, *p* < 0.0001). The present-day Swiss overall mean density of *Araneus diadematus* was roughly 140 times lower than the 20th century European overall mean (Table 1). (If the seven Swiss 20th century values (median = 0.0300 webs m^−2^; Table 1) were exclusively compared with the twenty Swiss 21st century values (median = 0.0000 webs m^−2^; Table 1), the difference between the two groups was still statistically significant at *p* < 0.05.)

Furthermore, the webs contained significantly fewer prey compared to previous studies (Mann–Whitney *U* test, n_1_ = 20, n_2_ = 18, Z = 4.5753, *p* < 0.0001; Table S2). (These figures refer to mean numbers of prey per web counted in mid-afternoon as a proxy for the daily prey capture rate of large orb-weaving spiders (see Supplementary Materials; Table S2).) 

## 4. Discussion

This study revealed that the large orb-weaving spider *Araneus diadematus* occurred in extraordinarily low population densities in the Swiss midland in 2019 (Table 1). *Araneus diadematus* spiders take down their webs during the night and rebuild them early in the morning. However, as laboratory experiments and field observations have revealed, not all individuals rebuild their webs the following day [30,36]. Some spiders, after consuming exceptionally large amounts of food at particularly favorable hunting sites, may cease feeding and rebuilding their webs for one or more days [36]. Thus, the percentage of spiders found with webs within an *A. diadematus* population is, in general, lower than 100% and usually in the range of 66% to 88%, depending on the availability of food at a particular time and location [30,36]. The question arises of whether the method of web counting applied in this study might have resulted in underestimation of the true population densities by overlooking a certain percentage of satiated spiders which had not rebuilt their webs at the time of the surveys. On this issue, it may be noted that densities assessed in the past century were for the most part also based on web counts [18,20,21,28,29,30,31,32,33,35], so the same methodological bias was involved at both time periods. Furthermore, since unsatiated spiders tend to rebuild their webs more frequently than fully satiated spiders [36,37], and since present-day spiders living in the era of insect loss are more likely to be unsatiated compared with half a century ago (Table S2), the frequency of web building nowadays might be expected to be rather higher. Thus, the possibility that the extraordinarily low present-day population densities were underestimations due to this particular type of methodological bias is highly unlikely.

In 2019, the webs appeared to be rather fragile compared with the stronger webs from previous decades, as is the case when malnourished spiders make webs with thinner threads (Nyffeler, pers. observations). This is in good agreement with laboratory experiments, in which the amount of thread produced is reduced if *Araneus diadematus* spiders are kept deprived of food [36] and it agrees with our observation that the webs contained significantly fewer prey compared to previous studies (Table S2). The overall impression gained during this survey was that there has been a paucity of prey throughout the entire study area in recent decades (Table S2), which has been confirmed by the observations of other researchers [2,38,39].

The abundance decline we show here of this once common spider in the Swiss midland is evidently revealing a bottom-up trophic cascade in response to prey scarcity recently documented in the same area, and more widely across western Europe [1,2,38,39]. The hypothesis that the availability of flying insect prey in the study area had drastically declined over the past decades is confirmed by the “windshield phenomenon” noticed throughout the Swiss midland (i.e., compared with previous decades, many times fewer flying insects are nowadays killed on the front windshields of cars [9,40]). This is in sharp contrast to the situation a few decades ago, when fairly frequent “wasteful killing“ (and, coupled with it, “partial consumption”) of insects in *Araneus diadematus* webs at favorable sites could be observed (with capture rates of sometimes up to 1000 prey web^−1^ day^−1^; [41]). The reduced food intake of *Araneus diadematus* in recent years (Table S2) is likely to have negatively impacted the fecundity and survival of this spider [42,43], which in turn may have led to the abundance decline documented in this study (Table 1). Sublethal effects of chemical pollution may have additional negative impacts on this spider’s survival [44,45,46,47,48,49].

Aside from food scarcity due to insect population declines and chemical pollution, other negative environmental developments have additionally contributed to the decreasing population densities of large orb-weaving spider species in the western European landscape. These include adverse effects of modern forestry practices (e.g., removal of forest understory and of shrubs at forest margins, large-scale aerial spraying for the purpose of caterpillar or bark beetle control), the transformation of traditional farmland into large-scale depauperate monocultures (accompanied by the loss of weedy field margins and hedges), the transformation of fallow grassland patches into construction sites, and the removal of shrubs and trees in urban and suburban gardens. That habitat conversion and degradation can have a strong detrimental effect on large orb-weaving spider abundance is well demonstrated by the example of the fate of a plot of land located near Zurich-Oberengstringen (in between Highway A3 and the Limmat riverbank). In the 1970s, tall grassland interspersed with shrubs covered this plot, and at that time, large orb-weaving spiders occurred there in high numbers (up to 6 m^−2^ in small, local patches [50]). During a visit to this site, 40 years later, it became apparent that the land had been converted to a highway rest area with short-cut lawns and, as a result, the density of large orb-weaving spiders had declined to a very low value of 0.002 m^−2^ (Nyffeler, pers. observations). Similar habitat conversions resulting in the destruction of suitable habitats for large orb-weaving spiders have been noted elsewhere.

We of course realize that the recorded declines were not based on systematic long-term monitoring and that they could be attributed to normal population cycling. However, while we are not here able to provide a smoking gun, several elements support a systematic decline. First of all, the dramatic decline up to < 1% of the reference baseline from 40 years ago has never been observed at shorter temporal time frames, and reaches far beyond the typical natural fluctuations in population sizes (e.g., [51]), even when they are known to be linked to resource pulses (e.g., [52]). Second, records from other locations show that this decline is occurring at such large spatial scales that it can only be explained by large-scale, more global environmental drivers like land-use change, climate change, or the global use of pollutants. Indeed, the extraordinarily low population densities of *Araneus diadematus* in the Swiss midland observed in the 2019 survey (Table 1) are supported by statements of biology students at the University of Basel, according to whom generally very few large orb-weaving spiders have been spotted in recent years during walks through forests and fields in the Basel region. Furthermore, in mid-June 2017, a group of over 40 biology experts conducted an extensive faunistic survey in the grounds of the Merian Gardens, a park-like area covering 180,000 m^2^ located on the outskirts of Basel [53]. In the course of this survey, only three specimens of *A. diadematus* could be found over a time period of 24 h suggesting that nowadays this once “abundant garden spider” [54] must have become rare in that area. The only large orb-weaver species still found in high densities during the 2019 survey in the Swiss midland was the bridge spider *Larinioides sclopetarius* (Nyffeler, pers. observations). This nocturnal species, which lives near water and frequently builds its webs on street lights and illuminated bridge railings, still gets sufficient amounts of food in the form of flying adults of semiaquatic insects (chironomids, ephemeropterans, trichopterans, etc.) attracted in large numbers to the artificial light [55,56,57]. Because of its capability to exploit these artificially high prey densities, *L. sclopetarius* is—in contrast to other large orb-weavers—extremely successful in colonizing urban habitats in high density even in the 21st century, not only in Switzerland, but also in other parts of western Europe [56].

Similar extraordinarily low population densities of *Araneus diadematus* (0.0004 adult females m^−2^ at a landscape scale) were also recorded in an extensive survey in nine landscapes of the Ghent region, northern Belgium, in the summer of 2014 [49]. Here, likewise, densities were one order of magnitude or more lower than those recorded one decade earlier (e.g., during a survey in 2004–2006, densities at two locations were 10–20 times higher compared to those ten years later [58]; Bonte, pers. observations). Interestingly, while other orb-weaver species—especially the larger ones—showed dramatic declines in both abundance and species richness along an urbanization gradient [59,60], *A. diadematus* was found to reach similar low densities across this land-use gradient. In other words, local densities at spatial scales of approx. 100 km were alarmingly low in both the more natural and human-impacted regions, demonstrating regional declines beyond the scale of local land use. Thus, extraordinarily low present-day population densities of *Araneus diadematus* have been recorded in two different regions of western Europe, ≈500 km apart. These declines extend beyond local environmental changes at small scales and suggest common negative impacts of intensive urbanization, climate change, or any other large-scale stressor across the entire landscape over the past half century [61,62].

The present study and that from northern Belgium suggest that the notion that *Araneus diadematus* is an abundant spider, as pointed out in much of the literature [63,64], has these days turned out to be a myth—at least in highly urbanized western European landscapes such as the Swiss midland and northern Belgium. So far, the apparent abundance decline of this species in western Europe has been ignored by the faunists in charge of compiling local Red Lists for spiders (e.g., [63,64]). Red Lists are based on the number of different locations within a landscape in which a species has been recorded, and not on absolute population densities. The fact that *Araneus diadematus* is still found in many places (although in highly reduced densities) may explain why it is still labeled an “abundant species” (e.g., [63,64]).

In contrast, present-day population densities of *Araneus diadematus* in some areas outside of continental western Europe still appear to be rather high. For instance, exterminators of the pest control firm Senske Services removed more than 75 *Araneus diadematus* spiders from the exterior of a “2500 square foot home“ (232.5 m^2^) in Seattle (USA) in August 2016 [65]. Evidently, the spiders at this particular location were still capturing sufficient food in the form of flying insects to enable them to build up a population density roughly 290 times that of the present-day Swiss overall mean density. Future detailed assessments of the population densities of this spider species in different parts of its geographic range are urgently needed, and may provide important information on the extent of the loss of flying insects in various regions of western Europe and further afield. We would in this respect like to promote the use of new citizen-science tools such as SpiderSpotter [66] to achieve these highly needed insights, and especially to guide short-term action to mitigate alarming declines in these sentinel arthropod top predators.

## 5. Conclusions

The drastic decline in the abundance of the orb-weaving spider *Araneus diadematus* over the past half-century documented in this study (Table 1) apparently reveals a bottom-up trophic cascade in response to the widespread insect losses that have occurred across large parts of Europe in recent decades [1,2,4,6,9,39,67,68]. There is evidence that other groups of aerial web-spinning spiders, which likewise depend on flying insects as food [17,18,19,21,69,70,71], have also become much rarer over the recent past (Nyffeler, pers. observations). So, for instance, the mesh-web weaver *Dictyna uncinata* (together with other dictynid species), whose small, tangled webs were found in large numbers on the leaves of garden plants a few decades ago [71], has apparently become very rare these days (Nyffeler, pers. observations). The ongoing abundance decline of the spiders parallels the dramatic abundance declines in other insectivorous animals such as insectivorous birds, bats, frogs, and lizards documented in recent decades [72,73,74]. 

To sum up, the findings of this study support the notion by other researchers that we now live in the midst of an ecological crisis in which trophic webs are being eroded and degraded as a result of adverse, man-made environmental impacts [13,67,73,75,76]. If this disastrous trend cannot be halted or even reversed in the very near future, “entire ecosystems will collapse due to starvation” [16].

## Figures and Tables

**Table 1:** Population densities of adult female *Araneus diadematus* (18 historical vs. 20 recent values). *Adults/subadults in September.

Habitat Type	Geographic Region	Period of Investigation	Spiders m^−2^	Source
Historical data *				
Fallow grassland, plot 1	Switzerland	1979	0.000	[21]
Fallow grassland, plot 2	Switzerland	1979	0.030	[21]
Fallow grassland, plot 3	Switzerland	1979	0.070	[21]
Fallow grassland, plot 4	Switzerland	1979	0.000	[21]
Shrubs	Switzerland	1979	0.930	[21]
Garden	Switzerland	1974–75	0.064	[20]
Glasshouses botanical garden	Switzerland	1932	0.001	[27]
Fallow grassland	Germany	1990	0.138	[18]
Fallow grassland	Germany	1998	0.056	[28]
Fallow grassland	France	1984	0.023	[29]
Hedgerow/grassland	France	1977	0.135	[30]
Hedgerow	Italy	1990–91	0.0005	[31]
Pine stand	Poland	1979	0.076	[32]
Clearing-forest ecotone	Poland	1979	0.320	[32]
Scotch pine wood	Netherland	1946	0.130	[33]
Oak wood	Netherland	1946	0.400	[33]
Oak stand	UK	1954–56	0.410	[34]
Heathland	UK	1968	0.024 *	[35]
Overall mean			0.1560	
Median			0.0670	
IQR			0.0175–0.1835	
**Recent data**				
Forest road	Northeastern Switzerl.	2019	0.002	This paper
Suburb	Zurich region, Switzerl.	2019	0.000	This paper
Suburb	Zurich region, Switzerl.	2019	0.000	This paper
Suburb	Northwestern Switzerl.	2019	0.001	This paper
Hedgerow + shrubs along a river bank	Zurich region, Switzerl.	2019	0.002	This paper
Forest edge + suburb	Northwestern Switzerl.	2019	0.002	This paper
Forest edge	Northwestern Switzerl.	2019	0.000	This paper
Forest road	Northwestern Switzerl.	2019	0.000	This paper
Garden + shrubs	Northwestern Switzerl.	2019	0.000	This paper
Graveyard shrubs	Northwestern Switzerl.	2019	0.000	This paper
Forest road	Northwestern Switzerl.	2019	0.000	This paper
Forest edge	Northwestern Switzerl.	2019	0.000	This paper
Forest edge	Northwestern Switzerl.	2019	0.000	This paper
Graveyard shrubs	Northwestern Switzerl.	2019	0.005	This paper
Public park	Northwestern Switzerl.	2019	0.000	This paper
Hedgerow	Northwestern Switzerl.	2019	0.000	This paper
Hedgerow	Northwestern Switzerl.	2019	0.000	This paper
Hedgerow + reedbelt	Central Switzerl.	2019	0.000	This paper
Garden	Northeastern Switzerl.	2019	0.006	This paper
Garden	Northwestern Switzerl.	2019	0.003	This paper
Overall mean			0.0011	
Median			0.0000	
IQR			0.0000–0.0020	

## Supplementary Materials

The following are available online at https://www.mdpi.com/2075-4450/11/4/248/s1, Table S1: Area size (m^2^) of the study plots, Table S2: Mean number of prey per web counted in mid-afternoon as a proxy for the daily prey capture rate of large orb-weaving spiders in western European habitats: Historical values vs. present day values.
