# Supplementary material for: Where Have All the Spiders Gone? Observations of a Dramatic Population Density Decline in the Once Very Abundant Garden Spider, Araneus diadematus (Araneae: Araneidae), in the Swiss Midland
Source: Insects. 2020 Apr 15;11(4):248. doi: 10.3390/insects11040248 (PMC7240396; doi:10.3390/insects11040248)
Supplement: Supplementary file 1 [file insects-11-00248-s001.pdf]

Supplementary Material:

Table S1. Area size (m^2) of the study areas

Habitat type	Geographic region	Plot size
**Historical data**		
Fallow grassland, plot 1	Canton Zurich, Switzerl.	~1000 m^2
Fallow grassland, plot 2	Canton Zurich, Switzerl.	~1000 m^2
Fallow grassland, plot 3	Canton Zurich, Switzerl.	~1000 m^2
Fallow grassland, plot 4	Canton Zurich, Switzerl.	~1000 m^2
Shrubs	Canton Zurich, Switzerl.	~1000 m^2
Organic garden	Canton Zurich, Switzerl.	450 m^2
Glasshouses Botanical Garden Bern	Canton Bern, Switzerl.	965 m^2
Fallow grassland	Germany	4 x 625 m^2
Fallow grassland	Germany	90 m^2
Fallow grassland	France	3300 m^2
Hedgerow/grassland	France	~400 m^2 *
Hedgerow	Italy	2000 m^2
Pine stand	Poland	100 m^2
Clearing-forest ecotone	Poland	100 m^2
Scotch pine wood	Netherland	157.5 m^2 **
Oak wood	Netherland	17.5 m^2
Oak stand	UK	2500 m^2
Heathland	UK	1450 m^2
**Present-day data (2019)**		
Forest road, Degersheim	Canton St. Gallen.	1000 m x 1 m
Suburb, Hönggerberg	Canton Zurich	1000 m x 1 m
Suburb, Zürichberg	Canton Zurich	1000 m x 1 m
Suburb, Rheinfelden	Canton Aargau	1000 m x 1 m
Hedgerow + shrubs along a river bank, Oberengstringen	Canton Zurich,	1000 m x 1 m
Forest edge + suburb, Arlesheim	Canton Baselland	500 m x 1 m
Forest edge, Münchenstein	Canton Baselland	500 m x 1 m
Forest road, Leymen	Alsace, France***	500 m x 1 m
Organic garden + shrubs, Dornach	Canton Solothurn	500 m x 1 m
Graveyard shrubs, Oberwil	Canton Baselland	500 m x 1 m
Forest road, Reinacherheide	Canton Baselland	200 m x 1 m
Forest edge, Bottmingen	Canton Baselland	200 m x 1 m
Forest edge, Rheinach	Canton Baselland	200 m x 1 m
Graveyard shrubs, Binningen	Canton Baselland	200 m x 1 m
Public park, Riehen	Canton Basel	200 m x 1 m
Hedgerow, Reinach	Canton Baselland	200 m x 1 m
Hedgerow, Schönenbuch	Canton Baselland	200 m x 1 m
Hedgerow + reedbelt, Sempach	Canton Luzern	200 m x 1 m
Organic garden, Flawil	Canton St. Gallen	800 m^2
Organic garden, Himmelried	Canton Solothurn	330 m^2

Additional information to Table S1

* Roughly estimated based on information (i.e., map of web distribution) found in Le Berre, M.; Ramousse, R.; Le Guelte, L. Eco-éthologie des Argiopidae: 1. Evolution temporelle d'une population d'*Araneus diadematus* Clerck dans son milieu naturel. *Atti. Soc. Toscana Sci. Nat. P. V. Mem. Ser. B* **1981**, *88*, 72-83.

** 20.5 ind/(9 x 17.5 m^2) = 20.5 ind/157.5 m^2 = 0.130 ind/m^2

*** Location in France, only 1.5 km from the Swiss border (Basel region)

Table S2. Mean number of prey per web counted in mid-afternoon as a proxy for the daily prey capture rate of large orb-weaving spiders in western European habitats: Historical values vs. present day value. References found in the paper.

Spider species	Habitat type	Year of investigation	$\bar{x}$ prey web^{-1}	Reference
**<u>Historical data:</u>**				
Araneus diadematus	Grassland, September	1979	13.5	Nyffeler 1982
Araneus diadematus	Grassland, August	1990	4.3	Malt 1996
Araneus diadematus	Grassland, September	1990	9.2	Malt 1996
Araneus diadematus	Maize field margins, July/August	2003	13.5	Ludy 2007
Araneus quadratus	Grassland, July	1979	21	Nyffeler 1982
Araneus quadratus	Grassland, August	1979	14	Nyffeler 1982
Araneus quadratus	Grassland, September	1979	20	Nyffeler 1982
Araneus quadratus	Grassland, August	1990	7.3	Malt 1996
Araneus quadratus	Grassland, September	1990	36.4	Malt 1996
Araneus marmoreus	Grassland	1981	14.1	Pasquet 1984
Argiope bruennichi	Grassland	1981	4.3	Pasquet 1984
Argiope bruennichi	Field margin, August/September	1976	13.2	Nyffeler & Benz 1978
Argiope bruennichi	Maize field margins, July/August	2003	4.9	Ludy 2007
Argiope bruennichi	Grassland, August	1990	6.5	Malt 1996
Argiope bruennichi	Grassland, September	1990	5.9	Malt 1996
Argiope bruennichi	Grassland, August	1979	10	Nyffeler 1982
Argiope bruennichi	Grassland, September	1979	10	Nyffeler 1982
Larinioides cornutus	Grassland, June/July	1979	18	Nyffeler 1982
Larinioides cornutus	Rye field	1976	15	Nyffeler 1982
Larinioides cornutus	Grassland, May/June	1961	6.7	Kajak 1965
**Overall mean**			**12.39**	
**Median**			**11.60**	
**IQR**			**6.55-14.77**	
**<u>Present-day data:</u>**				
Araneus diadematus	Diverse habitats August/September		1.00	This paper
**Overall mean**			**2.58**	
**Median**			**1.00**	
**IQR**			**0.75-2.50**	
